# Sense or nonsense? The role of protein acetylation in cyanobacterial photosynthesis and growth

**DOI:** 10.1093/plphys/kiad545

**Published:** 2023-10-12

**Authors:** Ryo Yokoyama

**Affiliations:** Assistant Features Editor, Plant Physiology, American Society of Plant Biologists; Max-Planck-Institute of Molecular Plant Physiology, Am Mühlenberg 1, Potsdam-Golm 14476, Germany

Posttranslational protein modification (PTM) has been studied as a regulatory mechanism to control a wide range of protein functions. Unlike irreversible PTMs (e.g. proteolysis), 1 or several specific amino acid residues of proteins are reversibly decorated by adding a chemically modifying group that potentially changes biochemical/biophysical protein properties, such as protein surface charge and structures, affecting various protein behaviors like enzyme activity, protein-protein interaction, and even protein localization (Friso and van Wijk 2015; Basak et al. 2016). Recent advances in mass spectrometry–based proteomics allow us to identify and quantify thousands of PTM sites in a more comprehensive manner, with a growing number of public databases of PTM sites available in various organism species (Doll and Burlingame 2015). According to dbPTM, a database of kingdam-wide PTM sites, phosphorylation, acetylation, and ubiquitination are the major PTMs, comprising more than 90% (∼827,000 sites out of ∼908,000) of all the reported PTMs (Li et al. 2022). Besides those, various types of PTMs were recently identified and characterized, such as nitrosylation and persulfidation (Li et al. 2022).

Do all these PTM sites play a regulatory role in protein characteristics? The answer is “no.” Even if you find PTM sites in your protein of interest, it does not mean that modifications of the protein at these sites have biologically meaningful impacts on protein activity or the biological processes you are focusing on. The effects of PTMs might not occur in vivo, or they might have little effect the protein properties (Basak et al. 2016). Therefore, further genetic and biochemical confirmation is needed to investigate how impactful each PTM site is for the protein function. A typical workflow in such a validation often starts with identifying enzymes mediating PTM at the amino acid residue of your interest, although this step is generally the hardest; very few examples where a specific modification enzyme that targets a site are known in vivo. Subsequently, phenotypic analyses of its loss-of-function mutant are conducted to elucidate how the disruption of PTM affects the PTM status, protein activity, and downstream biological functions. In planta site-direct mutagenesis that makes your protein constitutively active or inactive is further required to genetically examine whether the PTM at the site you are focusing on changes protein activity.

In this issue of *Plant Physiology*, Jia et al. (2023) outline an effective workflow for the characterization of protein acetylation at lysine (Lys, K) residues, one of the most conserved and widely used PTMs. Its best-known example is histone acetylation in eukaryotic cells by which gene expression and chromatin structure are reversibly regulated. Beyond nuclei, Lys acetylation occurs in various cellular compartments with diverse physiological functions in many model microbes, animals, and plants, including *Arabidopsis thaliana* (Wu et al. 2011; Drazic et al. 2016). The group had previously reported global Lys acetylome in cyanobacteria (Chen et al. 2017), the ancestor form of chloroplasts in eukaryotic photosynthetic organisms (Raven and Allen 2003). However, no acetyltransferase enzymes have ever been identified in cyanobacteria, which has been a bottleneck in understanding the role of Lys acetylation in cyanobacteria.

Jia et al. used the genomic information and *Escherichia coli* mutant complementation system to identify the cyanobacterial acetyltransferase Gcn5-related N-acetyltransferase (cGNAT2) in *Synechococcus* sp. PCC 7002 (Syn7002). The loss-of-function mutant of cGNAT2 exhibited limited growth capacity and photosynthetic activity, with 548 Lys acetylation sites disrupted in the mutant. This result demonstrates that cGNAT2 acetylates 548 endogenous substrates in the Syn7002 proteome. Among them, the most critical acetylation sites were narrowed down to elucidate why the Δ*cGNAT2* mutant showed impaired growth and photosynthetic phenotypes. The gene ontology (GO) annotations of the cGNAT2-mediated acetylation sites were enriched in photosynthesis-related proteins, particularly in NAD(P)H dehydrogenase (NDH)-1 complex enzymes, which mediates respiration, cyclic electron flow around photosystem I, and CO_2_ uptake in cyanobacteria (Battchikova et al. 2011). Thanks to this information, K89 of NdhJ, a hydrophilic arm subunit of the NDH-1 complex, was confirmed to be acetylated by cGNAT2 in vitro and in vivo. The Syn7002 strain carrying the K89R mutation, which is constitutively unacetylated, displayed severe growth and photosynthetic phenotypes that were comparable with the Δ*cGNAT2* and Δ*NdhJ* mutants. In contrast, the acetylation-mimicking K89Q mutant had identical phenotypes to the wild-type strain. Collectively, these findings identify cGNAT2 as a new acetyltransferase that mediates cyanobacterial growth and photosynthesis via the acetylation of more than 500 Lys, including the NdhJ K89 residue (Fig. 1).

**Figure 1.**
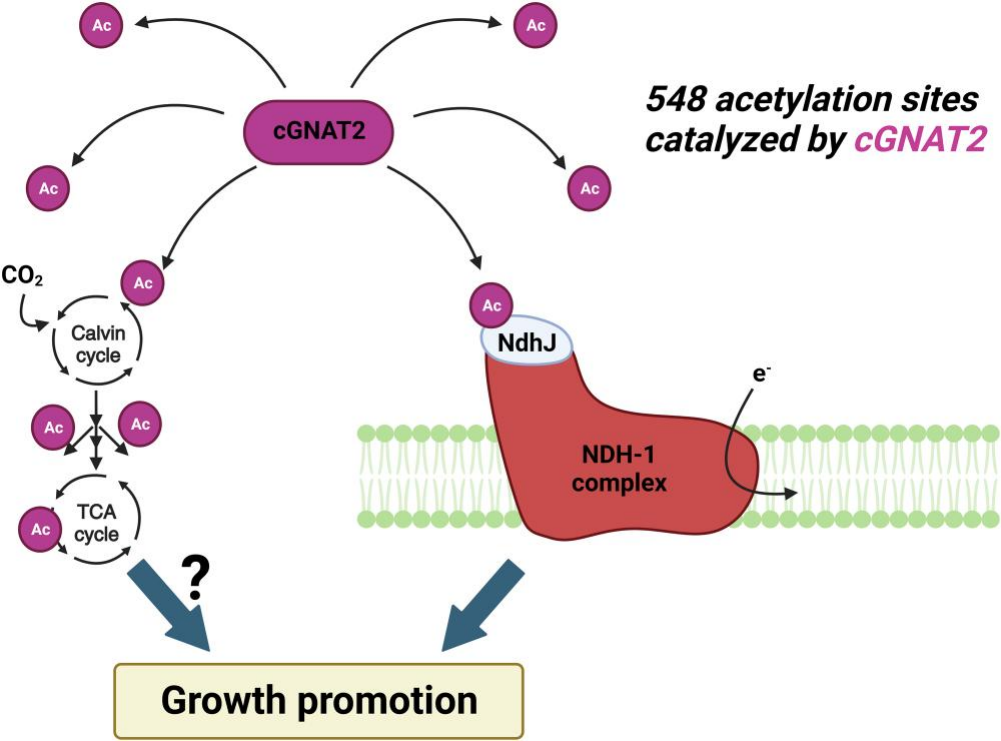
The role of cGNAT2 in the regulation of the NDH complex and cell growth. Besides acetylation at the K89 site of NdhJ, cGNAT2 mediates acetylation at other 547 Lys sites in the Syn7002 proteome, including enzymes involved in central carbon metabolism. However, this global acetylation information is not sufficient to conclude that these acetylation sites are influential enough to alter protein activity and cellular functions.

This study is a first step to understanding the role of acetylation in the regulation of growth and photosynthesis in cyanobacteria, yet it raises several open questions that will need to be tackled in the future. These future tasks include resolving the role of the other 547 Lys residues out of 548 cGNAT2-mediated acetylation positions identified in this study. Beyond the proteins involved in electron transport, Jia et al. also focused on numerous acetylation sites on enzymes that participate in central carbon metabolism, including the Calvin cycle, pentose phosphate pathway, glycolysis, and tricarboxylic acid cycle, which critically affect cell growth. However, this acetylation information is not sufficient to conclude that cGNAT2 regulates the enzyme activities of primary carbon metabolism (Fig. 1). Here, in vitro and in vivo mutagenesis that substitutes the Lys residues with arginine (R) or glutamine (Q) to mimic an unacetylated or acetylated status, respectively, will be necessary to test this hypothesis. Another open question is the evolutionary aspect of Lys acetylation in relation to the cyanobacterial origins of chloroplasts (Raven and Allen 2003). Sequence comparisons suggest that cGNAT2 has little similarity to other prokaryotic Lys acetyltransferases and instead appears closer to plant-type acetyltransferases, notably across the 5 residues critical for acetyltransferase activity. So, how is the role of Lys acetylation conserved in downstream biological functions in cyanobacteria and chloroplasts, among which are the roles of NDH complex and other carbon assimilation pathways? A possible answer may provide insights into the universality and diversity of protein acetylation during plant evolution.

## Dive Curated Terms

The following phenotypic, genotypic, and functional terms are of significance to the work described in this paper:

PTM AmiGo: PO:0005039

NADPH CHEBI: CHEBI:16474
